# Differential prevalence and geographic distribution of hepatitis C virus genotypes in acute and chronic hepatitis C patients in Vietnam

**DOI:** 10.1371/journal.pone.0212734

**Published:** 2019-03-13

**Authors:** Chau Le Ngoc, Thanh Tran Thi Thanh, Phuong Tran Thi Lan, Trinh Nguyen Mai, Trang Nguyen Hoa, Ngoc Nghiem My, Tan Le Van, Hung Le Manh, Phuong Le Thanh, Chau Nguyen Van Vinh, Guy Thwaites, Graham Cooke, Gabrielle M. Heilek, Cecilia Shikuma, Thuy Le, Stephen Baker, Motiur Rahman, on behalf of the VIZIONS consortium

**Affiliations:** 1 Oxford University Clinical Research Unit, Ho Chi Minh City, Vietnam; 2 The Hospital for Tropical Diseases, Ho Chi Minh City, Vietnam; 3 Centre for Tropical Medicine and Global Health, Nuffield Department of Clinical Medicine, Oxford University, Oxford, United Kingdom; 4 Imperial College, London, United Kingdom; 5 Roche Molecular Systems, Inc., Pleasanton, California, United States of America; 6 Hawaii Center for AIDS, University of Hawaii at Manoa, Honolulu, HI, United States of America; 7 Duke University School of Medicine, Durham, NC, United States of America; Centers for Disease Control and Prevention, UNITED STATES

## Abstract

**Background:**

The highest burden of disease from hepatitis C virus (HCV) is found in Southeast Asia, but our understanding of the epidemiology of infection in many heavily burdened countries is still limited. In particular, there is relatively little data on acute HCV infection, the outcome of which can be influenced by both viral and host genetics which differ within the region. We studied HCV genotype and IL28B gene polymorphism in a cohort of acute HCV-infected patients in Southern Vietnam alongside two other cohorts of chronic HCV-infected patients to better understand the epidemiology of HCV infection locally and inform the development of programs for therapy with the increasing availability of directly acting antiviral therapy (DAAs).

**Methods:**

We analysed plasma samples from patients with acute and chronic HCV infection, including chronic HCV mono-infection and chronic Human Immunodeficiency Virus (HIV)-HCV coinfection, who enrolled in four epidemiological or clinical research studies. HCV infection was confirmed with RNA testing. The 5’ UTR, core and NSB5 regions of HCV RNA positive samples were sequenced, and the genotype and subtype of the viral strains were determined. Host DNA from all HCV positive patients and age- and sex-matched non-HCV-infected control individuals were analysed for IL28B single nucleotide polymorphism (SNP) (rs12979860 and rs8099917). Geolocation of the patients were mapped using QGIS.

**Results:**

355 HCV antibody positive patients were analysed; 54.6% (194/355) and 46.4% (161/355) were acute and chronic infections, respectively. 50.4% (81/161) and 49.6.4% (80/161) of chronic infections had HCV mono-infection and HIV-HCV coinfection, respectively. 88.7% (315/355) and 10.1% (36/355) of the patients were from southern and central regions of Vietnam, respectively. 92.4% (328/355) of patients were HCV RNA positive, including 86.1% (167/194) acute and 100% (161/161) chronic infections. Genotype could be determined in 98.4% (322/328) patients. Genotypes 1 (56.5%; 182/322) and 6 (33.9%; 109/322) predominated. Genotype 1 including genotype 1a was significantly higher in HIV-HCV coinfected patients compared to acute HCV patients [43.8% (35/80) versus 20.5% (33/167)], (*p* = <0.001), while genotype 6 was significantly higher in chronic HCV mono-infected patients [(44.4% (36/81) versus 20.0% (16/80)] (*p* = < 0.004) compared to HIV-HCV coinfected patients. The prevalence of IL28B SNP (rs12979860) homozygous CC was 86.46% (83/96) in control individuals and was significantly higher in acutely-infected compared to chronically-infected patients [93.2 (82/88) versus 76.1% (35/46)] (*p* = < 0.005).

**Conclusion:**

HCV genotype 6 is highly prevalent in Vietnam and the high prevalence in treatment naïve chronic HCV patients may results from poor spontaneous clearance of acute HCV infection with genotype 6.

## Introduction

Hepatitis C virus (HCV) infection is a major public health threat, and the Global Health Sector Strategy (GHSS) on viral hepatitis 2016–2021 calls for the elimination of viral hepatitis, reducing new infections by 90% and mortality by 65% by 2030 [1]. The World Health Organization (WHO) estimates that approximately 71 million people are suffering from chronic HCV infection globally, and annually 700,000 people die from HCV-related complications, including liver cirrhosis and hepatocellular carcinoma (HCC) [1]. African continent have highest HCV disease burden followed by Asia (2.8% and 2.7% seroprevalence respectively) [2]. One fifth of the global HCV burden (14/71 million) is in Western Pacific Region which includes Vietnam [1]. HCV transmission results from direct exposure to contaminated blood and is associated with injectable drug use (IDU), iatrogenic exposure (blood transfusion, surgical and dental procedures, dialysis, acupuncture, needle stick injury, and use of unsterilized needles), body piercing and less frequently, through vertical transmission and high risk sexual behavior [3, 4]. Acute HCV infection is infrequently diagnosed as the majority of acutely infected individuals are asymptomatic [5]. After acute infection, approximately 75–85% of patients do not clear the virus by six months and develop chronic HCV infection [5]. Progression to chronic HCV infection depends on several factors, including the age at the time of infection, gender, ethnicity, host genetic factors, immune status, development of jaundice during acute infection and viral genotype and subtype [6]. It is estimated that chronic HCV infection accounts for 20% of HCC globally [7]. HCV related HCC is more prevalent in countries where HCC prevalence is low (Western Europe and North America) or intermediate (Japan, Italy and Spain) [7–10].

HCV is classified into seven genotypes 1–7; each genotype is further divided into a variable number of more closely related subtypes (currently more than 100 subtypes) [11]. The natural course of infection, pathogenesis, treatment regimens, treatment duration and outcomes largely depend on genotype and subtype of the infecting HCV strain [12, 13]. For examples, the rate of evolution to chronicity is higher in genotype 1b compared to other genotypes (92% versus 33–50%) [14]. A recent case control study has documented the association between genotype 1b and HCC and the multivariate adjusted odds ratio of developing HCC for HCV genotype 1b compared to non-1b was 1.65 (95% confidence interval (CI); 1.16–2.33) [15]. Different transmission routes for HCV infection has shown to be associated with the genotype of the virus. Subtype 1b transmits effectively via blood transfusion, while subtypes 1a and 3a transmit predominantly through IDU [16].

Besides genotype, host factors such as single-nucleotide polymorphisms (SNP) of IL28B (rs12979860 and rs8099917) in the upstream of *IL28B* gene on chromosome 19 have been shown to be associated with spontaneous clearance of acute HCV infection, jaundice during acute infection and sustained viral response (SVR) on pegINF/ribavirin(RBV) treatment [17, 18]. Patients who are homozygous carriers of cytosine (CC) at position rs12979860, have a much higher likelihood of spontaneous clearance compared to homozygous thiamine TT or heterozygous CT [(64%; 43/67) versus (6%; 2/33) and (24%; 22/90)] respectively (*p*<0.001). Jaundice during acute infection is more common among patients with CC genotype than non-CC patients (CT or TT) (32.7% versus 16.1%) [18].

HCV genotypes have distinct geographic distribution. Genotype 6 is more prevalent in Southeast Asian countries [19], including Southern China, Myanmar, Laos, Vietnam and Cambodia [2, 20–22]. Genotype 6 is the most diverse with 31 subtypes currently recognized and has shown to have emerged and evolved in Southeast Asia [23]. Genotype 6 has also identified in industrialized countries such as Canada and Australia from a Southeast Asian linage due to population immigration [24].

Vietnam is amongst the 20 countries with the highest burden of HCV in the world with an estimate of 1.5 million chronic HCV infected people (estimated HCV RNA prevalence 1.1%) (https://www.who.int/westernpacific/health-topics/hepatitis/regional-hepatitis-data). The prevalence of positive HCV antibody among the general population is between 1% and 4.7% [25–28], but is substantially higher among HIV-infected individuals (22.9–89.0%), IDUs (74.0% - 87.0%), and multi transfusion and dialysis patients (6.0–26.6%) [23, 29–32]. Vietnam has recently revised its national guidelines for the treatment of HCV infection, and access to affordable generic DAAs for HCV infection is rapidly expanding. However, knowledge of the molecular epidemiology of HCV infection and the host genetic diversity among HCV infected populations in Vietnam are lacking. In order to inform the development of HCV treatment programs in country, we analyzed HCV RNA positive samples from four epidemiological and clinical studies in Vietnam. Our specific aims were to i) determine the differential prevalence and geographical distribution of HCV genotypes and subtypes in acutely HCV infected and chronically HCV-infected patient populations ii) determine the differential prevalence of IL28B SNP (rs12979860 and rs8099917) among acutely HCV-infected and chronically HCV-infected patient populations in Vietnam.

## Materials and methods

### Ethics statement

All studies were approved by ethical review committee of the Hospital for Tropical Diseases (HTD) in Ho Chi Minh City (HCMC) and Oxford Tropical Research Ethics Committee (Table 1). Written informed consent was mandatory for entry into all the studies, written informed consent was requested from a parent or guardian from those aged <16 years.

**Table 1:** Study title, ethical approval, inclusion/exclusion criteria, study sites, study duration, sample size, number (percentage) of HCV positive patients and types of HCV infection.

SL No	Study title	OxTREC approval	HTD approval	Inclusion & exclusion criteria	Study site	Study period	Sample size	HCV positive n(%)	HCV infection status
1	Epidemiology and etiology of viral hepatitis and molecular characterization of Hepatitis B and C virus isolated from patients attending at Hospital for Tropical Diseases (HTD) in Ho Chi Minh City (HCMC), Vietnam.	OxTREC; 152–12	SC/ND/12/14	**Inclusion criteria:** • symptom consistent with acute viral hepatitis (e.g., fever, headache, malaise, anorexia, nausea, vomiting, diarrhea, AND abdominal pain • aged ≥1 year • written informed consent, • total bilirubin ≥50 mmol/l or ≥3 mg/dl and/or • ALT ≥200 international unit **Exclusion criteria** • previously diagnosed as having hepatitis of any etiology • previous enrolment in this study, • known to have liver cirrhosis, hepatocellular carcinoma, or other terminal illness, and • alcoholic hepatitis, or drug or toxin induced hepatitis.	1.Hospital for Tropical diseases; Ho Chi Minh City,	Jan 2015 to May 2016	737	96 (13.0)	Acute infection
2	The etiology of diarrhea, hepatitis, respiratory infections and central nervous system infections in provincial hospitals in Vietnam	OxTREC; 15–12	SC/ND/13/28	**Inclusion criteria:** • symptom consistent with acute viral hepatitis (e.g., fever, headache, malaise, anorexia, nausea, vomiting, diarrhea, and abdominal pain • aged ≥1 year • written informed consent, • total bilirubin of ≥50 mmol/l or ≥3 mg/dl and • ALT ≥200 international unit (IU)/liter (L). **Exclusion criteria:** • previously diagnosed as having hepatitis of any etiology • previous enrolment in this study, • known to have liver cirrhosis, hepatocellular carcinoma, or other terminal illness, and • alcoholic hepatitis, or drug or toxin induced hepatitis.	1. Hospital for Tropical diseases; Ho Chi Minh City, 2. National Hospital for Tropical Diseases, Hanoi 3. Hue provincial hospital, Hue 4. Khanh Hoa provincial hospital, Khanh Hoa 5. Dak Lak provincial hospital, Dak Lak 6. Dong Nai provincial hospital, Dong Nai 7. Dong Thap provincial hospital, Dong Thap	Jan 2013 to Oct 2016	1089	98 (8.9)	Acute infection
3	Molecular characterization of Hepatitis B and C virus isolated from patients attending at Hospital for Tropical Diseases (HTD) in Ho Chi Minh City (HCMC), Vietnam.	OxTREC; 5–12	SC/ND/12/4	**Inclusion criteria:** • No symptom consistent with acute viral hepatitis • previously diagnosed as hepatitis C positive • HCV RNA positive for at least 1 year • aged ≥1 year • written informed consent **Exclusion criteria** • previous enrolment in this study, • known to have liver cirrhosis, hepatocellular carcinoma, or other terminal illness, and • alcoholic hepatitis, or drug or toxin induced hepatitis.	1.Hospital for Tropical diseases; Ho Chi Minh City,	Jan 2012 to December 2014	81	81 (100)	Chronic infection
4	Hepatic Safety of Raltegravir-based and Efavirenz-based Antiretroviral Regimens in Antiretroviral-Naïve HIV-infected Subjects Co-Infected with Hepatitis C	OxTREC- 36–10	CS/ND/10/15	**Inclusion criteria:** • HIV infected patients, age ≥18 years, meet Vietnam guideline to begin ART (CD4 count < 350 cells/mm^3^ and/or WHO stage III or IV disease) • Hepatitis C infection as documented by positive HCV antibodies and a detectable serum HCV RNA level • AST and ALT ≤ 2 x ULN (≤ 80 U/L) • Estimated creatinine clearance ≥ 60 mL/min (Calculation by Cockcroft and Gault: (140 –age in years) × (body weight in kg) ÷ (serum creatinine in mg/dL × 72). For women, multiply by 0.85 **Exclusion criteria:** • Any prior ART • Positive Hepatitis B surface antigen • Clinical evidence of de-compensated cirrhosis (ascites, encephalopathy, esophageal bleeding) • Requirement for acute therapy for other AIDS-defining illness within 14 days prior to study entry • Currently on rifampicin therapy • In the first trimester of pregnancy, intent to become pregnant, or breast feeding during the study period	1.Hospital for Tropical diseases; Ho Chi Minh City,	June 2013 to June 2017	80	80 (100)	Chronic infection (HIV-HCV co-infection)

HTD; Hospital for Tropical diseases

### Case definition

HCV infection in this study was defined as per the Council of State and Territorial Epidemiologists (CSTE) case definition, (https://wwwn.cdc.gov/nndss/conditions/hepatitis-c-probable acute/case-definition/2016/, https://wwwn.cdc.gov/nndss/conditions/hepatitis-c-chroniCCase-definition/2016/). Briefly, the case definition for acute HCV infection is defined as having a discrete onset of symptoms consistent with acute hepatitis (e.g., nausea, anorexia, fever, malaise, or abdominal pain) AND either jaundice or elevated serum aminotransferase levels >200 IU/L during the period of acute illness AND a positive HCV RNA amplification test. A case definition for chronic HCV infection is defined as having no clinical or laboratory evidence indicative of acute hepatitis (above) AND a positive HCV antibody test AND a positive HCV RNA test.

### Study population

Plasma samples from 355 HCV positive (either HCV antibody or antigen or HCV nucleic acid test (NAT) positive) patients recruited in four epidemiological and clinical research studies conducted in Vietnam from 2012 to 2016 were included in this study. Information about the study title, ethical approval, inclusion and exclusion criteria, enrollment sites, study duration, sample size, number of HCV positive samples and types of HCV patients are presented in Table 1. Among the HCV positive patients, 194 were classified as acute infection, and 161 as chronic infection including 81 chronic HCV mono-infection and 80 HIV-HCV coinfection. These studies were conducted in seven provincial hospitals across Vietnam. In addition, plasma samples from ninety-six age (± 5 years) and sex matched HCV-negative patients attended to the Hospital for Tropical Diseases (HTD) between 2013 and 2016 were used as control group for the IL28B SNP analysis. Sociodemographic data, geolocation information (province, district, and city), clinical data, along with laboratory data on hematology, chemistry (aspartate aminotransferase (AST) alanine aminotransferase (ALT), bilirubin, Gamma-glutamyl transpeptidase (GTT), alpha fetoprotein (AFP)), serological tests for hepatitis viruses were extracted from respective study database. Anti HCV antibody was detected by AiDTM anti-HCV ELISAPlus (Beijing Wantai Biological Pharmacy Enterprise Co., Ltd, China) test as per manufacturer specification (sensitivity 100%, specificity 99.96).

### HCV RNA detection and genotyping

Viral RNA was extracted from 140 μl of plasma using MagNa pure 96 DNA and viral NA small volume kit (Roche Diagnostics, Basel). Viral RNA was reverse transcribed using superscript III reverse transcriptase protocol as described earlier [22]. The resulting cDNA was used for amplification of 236 bp 5’UTR, 377 bp NSB5 and 464 bp core region using primers through nested PCR as described earlier [22]. Amplicons were run on 1.5% agarose gel (Merck, Germany) and purified using the Agincourt AMPure XP (Beckman Coulter, Inc) according to the manufacturer’s protocol. Purified amplicons were sequenced both forward and reverse direction using Big Dye Terminator Cycle Sequencing Ready Reaction Kit and Applied Biosystems 3130xl Genetic Analyzer (Applied Biosystems).

The nucleotide sequences were aligned and the consensus sequence was generated. This includes 236 bp 5’UTR (position 78 to 314), 464 bp core sequence (position 288 to 752) and 377 bp NS5B (position 8259 to 8636) of GenBank sequence AY051292. Forty seven well characterized HCV whole genome sequences representing all genotypes and subtypes were downloaded from GenBank and used as reference sequences [11]. The reference sequences and the sequences from the current study were subjected to phylogenetic analysis using Genious 8.0.5 software package (http://www.geneious.com). NSB5 sequences (if NS5B sequence is not available, core or 5’UTR sequences respectively) from the present study and the corresponding region of reference sequences were aligned with MUSCLE alignment program available within the Genious package. The sequence alignments were then subjected to Jmodel test to identify the best model for phylogenetic analysis [33]. The suggested nucleotide substitution model (GTR+G+I) was subsequently used in phylogenetic analysis using Neighbor-Joining method (available in Genious package). To confirm the reliability of phylogenetic tree analysis, bootstrap resampling and reconstruction were carried out 1000 times. All sequences from this study was submitted to GenBank (accession numbers for 5’UTR MH191406—MH191721; core region MH191722—MH192024 and, NS5B region MH192025—MH192337).

### IL28B polymorphism determination

96 controls and randomly selected 88 acute and 83 chronic (including 46 chronic mono-infections and 37 HIV-HCV coinfection) HCV patients’ samples were used for IL28B SNP assessment. DNA was extracted from stored cell pellets using the DNeasy blood and tissue kit (Qiagen, Germany) according to the manufacturer’s protocol. SNPs (rs8099917 and rs12979860) in IL28B gene were determined with the TaqMan SNP Genotyping assay (Applied Biosystems, Foster City, CA,). SNP at rs12979860 (CC, CT and TT genotypes), and rs8099917 (TT, GT and GG genotypes) were determined according to manufacturer’s protocol. SNP at rs12979860 (CC, CT and TT genotypes) and rs8099917 (TT, GT and GG genotypes) was compared between acute and chronic HCV patients and control group.

### Data analysis

All data extracted from the respective study database were analysed using Statistical Package for Social Science (SPSS) software (IBM SPSS Statistics 23, NY USA). The geo-location data of the patients were analyzed by GIS package version 2.18. Cases from northern regions of Vietnam were excluded from the geolocation analyses due to low patient numbers. Categorical variables were compared with Chi-square test and continuous variables by Student’s t-test. One way ANOVA was used to compare proportions between multiple groups. A *p* value <0.05 was regarded as statistically significant.

## Results

### Baseline data

A total of 355 HCV positive samples were available for analysis in this study. Among these, 54.6% (194/355), 22.8% (81/355) and 22.4% (80/355) were classified as acute HCV infection, chronic HCV mono-infection, and HIV-HCV coinfection, respectively. Among 194 acutely-infected HCV patients, 86.1% (167/194) was HCV RNA positive and were included for further analysis. All 161 chronically HCV-infected patients were HCV RNA positive. Overall, 92.4% (328/355) were HCV RNA positive only; 84.2% (299/355) of the patients were both HCV antibody and HCV RNA positive; and 91.8% (326/355) were HCV antibody positive only. 8.8% (29/328) of HCV RNA positive samples were HCV antibody negative, and 8.3% (27/326) of HCV antibody positive samples were HCV RNA negative.

The sociodemographic information of the patients are presented in Table 2. 59.3% (195/328) were male including the majority (87.5%; 70/80) of the patients in HIV-HCV coinfection group. The mean age of acute, chronic HCV mono-infected and HIV-HCV co-infected patients were 42 years, 48 years, and 35 years, respectively. Among all patients, 6.7% (24/355) were Hepatitis B surface antigen (HBs Ag) positive, including 10.8% (21/194), and 3.7% (3/81) in acute and chronic HCV mono-infection respectively.

**Table 2:** Socio demographic characteristics and prevalence of genotypes and subtypes of Hepatitis C PCR positive patients analyzed in the study.

		All patient	Acute infection	Chronic infection	HIV-HCV coinfection	P^3^	pᶞ
		100% (328)	50.9% (167)	24.7% (81)	24.4% (80)		
		% (n)	% (n)	% (n)	% (n)		
Age	0–20	1.2 (4)	2.4 (4)	0.0 (0)	0.0 (0)		
	21–30	16.2 (53)	23.4 (39)	6.2 (5)	11.3 (9)		
	31–40	36.6 (120)	26.9 (45)	13.6 (11)	80.0 (64)		
	41–88	46.0 (151)	47.3 (79)	80.2 (65)	8.8 (7)		0.000***
Gender	Male	59.3 (195)	48.8 (81)	53.1 (43)	87.5 (70)		
	Female	40.7 (133)	51.2 (85)	46.9 (38)	12.5 (10)		0.000***
Genotype ^b^	Subtype ^b^						
1		56.5 (182)	50.9 (82)	46.9 (38)	77.5 (62)	0.000***	
	1a	27.6 (89)	20.5 (33)	25.9 (21)	43.8 (35)	0.001**	
	1b	28.9 (93)	30.4 (49)	21.0 (17)	33.7 (27)	0.169	
2		7.5 (24)	9.9 (16)	8.6 (7)	1.3 (1)	0.048*	
	2a	4.0 (13)	5.0 (8)	4.9 (4)	1.3 (1)	0.346	
	2b	0.6 (2)	1.2 (2)	0.0 (0)	0.0 (0)	0.368	
	2m	2.8 (9)	3.7 (6)	3.7 (3)	0.0 (0)	0.218	
3		2.2 (7)	3.7 (6)	0.0 (0)	1.3 (1)	0.14	
	3a	0.9 (3)	1.9 (3)	0.0 (0)	0.0 (0)	0.222	
	3b	1.2 (4)	1.9 (3)	0.0 (0)	1.3 (1)	0.469	
6		33.9 (109)	35.4 (57)	44.4 (36)	20.0 (16)	0.004**	
	6a	18.3 (59)	18.6 (30)	19.8 (16)	16.3 (13)	0.84	
	6e	12.1 (39)	12.4 (20)	19.8 (16)	3.8 (3)	0.008**	
	6h	0.3 (1)	0.6 (1)	0.0 (0)	0.0 (0)	0.608	
	6k	0.3 (1)	0.0 (0)	1.2 (1)	0.0 (0)	0.226	
	6l	2.2 (7)	2.5 (4)	3.7 (3)	0.0 (0)	0.256	
	6p	0.6 (2)	1.2 (2)	0.0 (0)	0.0 (0)	0.368	

* p = 0.01–0.05

** p<0.001–0.01

*** p<0.001.

^b^ Data available for 322 patients (161 acute infection, 81 chronic infection and 80 HIV-HCV coinfection)

ᶞ p value between chronic HCV mono-infection and HIV-HCV coinfection determined by Chi square for nominal and Man Whitney test for continuous variables.

ᶾ p value between acute HCV, chronic HCV mono-infection and HIV-HCV coinfection as determined by one way ANOVA test

### Geolocation of patients

88.7% (315/355) of the patients were from the southern regions, including 60.5% (215/355) from the Southeast sub region, and 28.1% (100/355) from the Mekong Delta sub region. 10.1% (36/355) from the central regions including 3.9% (14/355) from the Central Highlands sub region, 1.4% (5/355) from the North Central Coast sub region, and 4.8% (17/355) from the South Central Coast sub region (S1 Table).

### HCV genotyping

**Fig 1:**
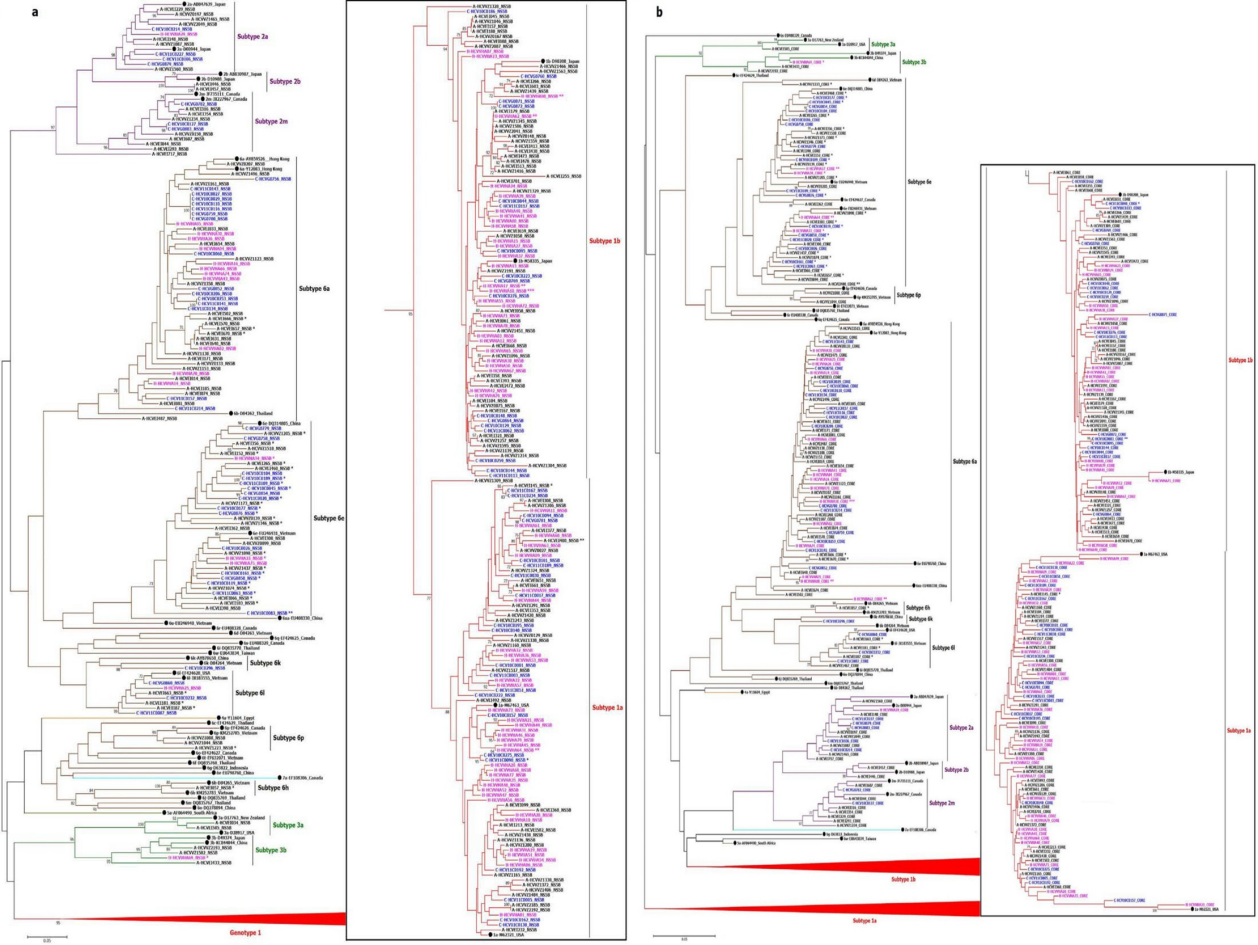
A midpoint rooted tree showing the relationship between the Vietnamese HCV (n = 322) sequences with 43 reference sequences downloaded from the GenBank database (all genotypes and selected subtypes). Tree was constructed using Neighbor-Joining method available in Genious software using GTR+G+I nucleotide substitution model with 1000 bootstrapping replicates. Bootstrap values greater than 70% is shown on the branch nodes. Acute, chronic mono-infection and HIV-HCV coinfection is presented by suffix A (in black text), suffix C (in blue text) and suffix H (in purple text) followed by the patient ID and sequence region respectively. The reference genomes are presented as subtype followed by Gene Bank accession number and country of origin. The reference genomes are highlighted with a filled dark circle. The scale bar indicates the number of nucleotide substitution. Red, purple, green, and brown color is used for genotype 1, 2, 3, and 6. (a) Analysis was based on a 377 bp HCV NS5B gene (position 8259 to 8636 relative to GenBank sequence AY051292). Sequences with discordant genotype between NS5B and 5 UTR, and NS5B and core is marked with * and ** respectively. (b) Analysis was based on a 464 bp core sequence (position 288 to 752) relative to GenBank sequence AY051292). Sequences which have discordant genotype between core and 5’UTR with NS5B is marked with ***.

HCV RNA positive samples (n = 328) were included for HCV genotype analysis. Any HCV RNA region (5’UTR, core, and NS5B) could be amplified and sequenced in 98.2% (322/328) of samples and all three regions could be amplified and sequenced in 87.8% (288/328) of the samples. The phylogenetic analysis using the NS5B and core region are presented in Fig 1. All Vietnamese isolates belong to four genotypes (1, 2, 3, and 6) and 13 subtypes (1a, 1b, 2a, 2b, 2m, 3a, 3b, 6a, 6e, 6h, 6k, 6l, and 6p) (Table 2). The overall prevalence of genotype 1, 2, 3 and 6 was 56.5% (182/322), 7.5% (24/322), 2.2% (7/322), 33.9% (109/322), respectively (Table 2). All genotype 1 isolates belong to subtype 1a or 1b. Among genotype 6 isolates, the majority were subtype 6a (54.1%; 59/109) and 6e (35.8%; 39/109). The prevalence of genotype 1a in HIV-HCV coinfection group was significantly higher than in acute or chronic HCV mono-infection groups, 43.8% (35/80) versus 20.5% (33/176) and 25.9% (21/81), respectively, (p<0.001, one-way ANOVA test). Genotype 6 was significantly higher in chronic HCV mono-infection patients compared to acute or HIV-HCV co-infection group (44.4%; 36/81 versus 33.4%;57/161 and 20%; 16/80 respectively) (p = 0.004, one-way ANOVA test).

**Fig 2:**
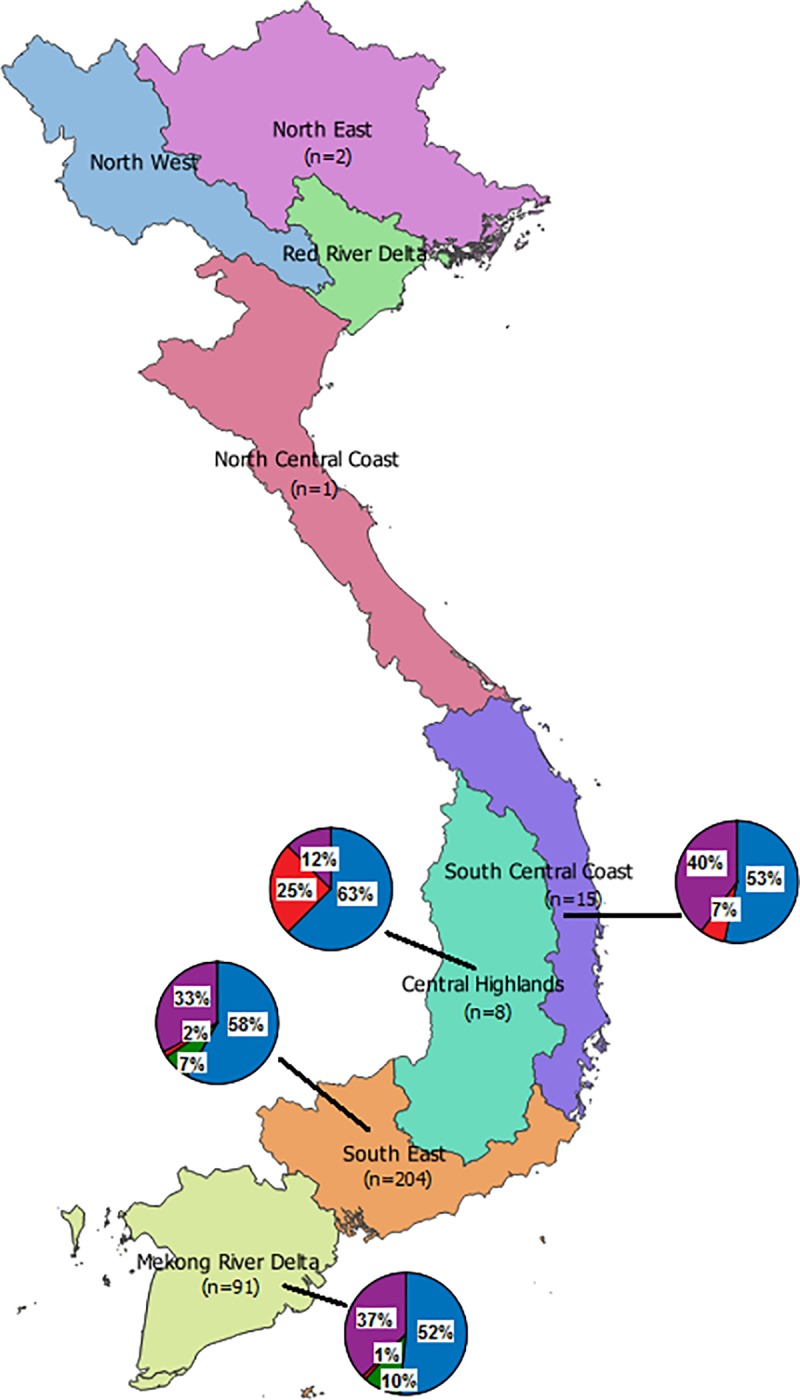
Geographical distribution of HCV genotypes in different sub regions of Vietnam. Sub regions are presented by different colors. Number of isolates and proportion of different genotypes in each sub region are presented in blue (genotype 1), green (genotype 2), red (genotype 3) and purple (genotype 6) colors.

The distribution of HCV genotypes among different geographic regions were analysed and presented in Fig 2. HCV genotype 1 accounted for more than half of isolates in all sub regions. More than one third of the viral strains from the southern regions were genotype 6, including 66.3% (67/101) in the South East and 33.7% (34/101) in the Mekong Delta sub region. 25% of isolates from the Central Highlands sub region was genotype 3. 10% (9/91) and 7.0% (15/204) of the viral strains in Mekong Delta and South east sub region was genotype 2.

We evaluated the concordance of genotype determination by each individual genetic region (NS5B, core and 5’UTR) in 288 strains with all three region sequenced. Discordant in genotypes was identified in 43 (14.9%; 43/288) viral strains. The discordance was observed in 36 strains (12.5%; 36/288) when the 5’UTR region based genotype was compared with the NS5B and core based genotypes (genotype 6e is being identified as genotype 1a or 1b). The discordance was observed in 6 (2.0%; 6/288) and one (0.3%; 1/288) strains when the core based genotype was compared with the NS5B and 5’UTR based genotype, and the NS5B based genotypes was compared with the core and 5’UTR based genotype respectively. Among the discordant genotype strains, 93.0% (40/43) was genotype 6 including 75.0% (30/40) subtype 6e (Fig 1A and 1B sequences with * asterisk mark). In 7 isolates (2.4%; 7/288), the genotypes defined by the core gene were different from that of the NS5B gene indicating a possible mixed infection (Fig 1A and 1B sequences with ** and *** asterisk mark). The homology of 5’UTR, core and NS5B sequence of these viral strains with the reference strains are presented in S2 Table. All possible mixed infection sequences belonged to genotype 1 or 6. Six of these seven viral strains were identified from chronically HCV-infected patients including five from HIV-HCV coinfected and one from HCV mono-infected patients. We further examined the diversity of the sequence within acute, chronic mon-infected and HIV-HCV co-infected patients. The diversity was higher among viral strains from acutely infected compared to chronically infected patients (NS5B; 92.3% similarity in acute strains versus 95.7% in chronic infection strains) (Fig 1A and 1B).

We examined the association between HCV genotypes and liver enzyme (AST, ALT, bilirubin, GTT, AFP) and HCV RNA measurement in acute, chronic HCV mono-infection and HIV-HCV coinfection. Liver enzyme and HCV RNA levels were significantly higher in acutely-infected patients compared to chronically-infected patients. No significant differences in the liver enzymes and HCV RNA levels were observed between the different genotypes within these three patient groups. All statistical comparisons are presented in the S3 Table.

### Prevalence of IL28B SNP among different groups

**Fig 3:**
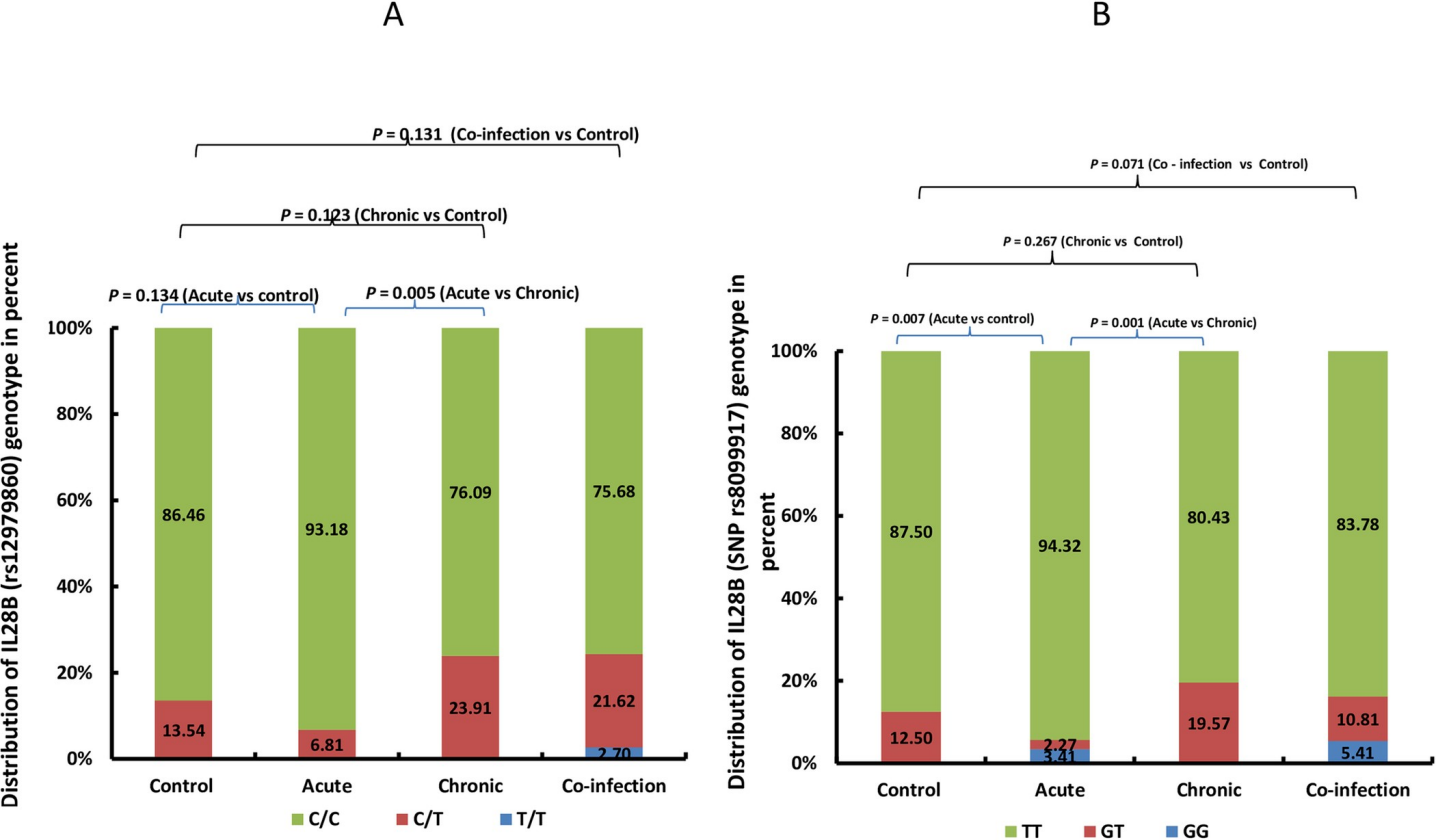
The prevalence of IL28B SNP (rs12979860 and rs8099917) in 171 patients. 88 acute, 46 chronic HCV mono-infection and 37 HIV-HCV coinfection and 96 age (±5 years) and sex matched non hepatitis control patients. The percent prevalence of SNPs is presented in Y axis and the patient groups and controls in X axis. P value was calculated using chi square and fisher’s exact test as appropriate. (A) The prevalence CC, CT and TT for rs8099917. The proportion of CC, CT and TT alleles are presented in green, orange and blue color bars respectively. P value was calculated between control and all groups (acute HCV, chronic HCV mono-infection and HIV-HCV coinfection) individually and acute HCV and chronic HCV mono-infection group. (B) The prevalence TT, T/G and G/G for rs12979860. The proportion of TT, GT and GG alleles are presented in green, orange and blue color bars respectively. P value was calculated between control and all groups (acute HCV, chronic HCV mono-infection and HIV-HCV coinfection) individually and acute HCV and chronic HCV mono-infection group.

The IL28B SNP (rs12979860 and rs8099917) was analysed in 267 patients including 88, 46, 37 and 96 acute, chronic HCV mono-infection, HIV-HCV coinfection, and control patients respectively (Fig 3). For the rs12979860, 228 of 267 patients (85.39%, [95% CI, 81.03% to 89.55%]) were homozygous for CC, 1 (0.37% [95% CI, -0.35% to 1.09%]) were homozygous for TT and, 38 (14.23% [95% CI, 10.11% to 18.35%]) was heterozygous. There was no significant difference in CC prevalence between HCV-negative control group and acute HCV group (86.46%; 83/96 versus 93.18%; 82/88), *p* = 0.134, Chi Square test. However, the prevalence of CC allele was significantly higher in acute HCV patients compared to chronic HCV patients (93.18%; 82/88 versus 76.09%; 35/46), *p* = 0.002, Chi Square test (Fig 3A).

For the rs8099917, 235 of 267 patients (88.01% [95% CI, ±3.83; 84.18% to 91.84%]) were homozygous for TT, 5 (1.87% [95% CI, ± 1.62; 0.27% to 3.47%]) were homozygous for G/G and, 27 (10.11% [95% CI, ±3.56; 6.55% to 13.67%]) were heterozygous (G/T). There was significant difference in TT prevalence between control group and acute HCV patients (87.50%; 84/96 versus 94.32%; 83/88) p = 0.007, Chi Square test, and between acute and chronic HCV patients (94.32%; 83/88 versus 80.43%; 37/46), p = 0.001, Chi Square test (Fig 3B).

## Discussion

HCV pathogenesis depends on host and viral factors including host genetics, the genotype and subtypes of the infecting viral strain [34]. The prevalence of HCV genotype among IDUs, dialysis and multi-transfusion and HIV patients have been reported earlier; however, data on HCV genotype among the general population and especially in acute HCV infection are lacking in Vietnam [22, 35–42]. By collating data from four hospital based studies in Vietnam to identify the prevalence HCV genotype and to examine the geographic distribution of genotypes, we identified a high genetic diversity in HCV, illustrating a polyphyletic nature of the HCV epidemic in Vietnam. Genotype 1 was predominant, with nearly half of the acute and chronic HCV mono-infection and three fourth of the HIV-HCV coinfection groups. This is consistent with earlier studies in Vietnam, one of which documented a high (60.0%) prevalence of genotype 1 in IDUs [30]. HIV-HCV coinfected patients had the highest prevalence of subtype 1a (43.8% compared to 20.5% in acute and 25.9% in HCV monoinfected patients. Subtype 1a has been shown to be common in male with high risk behaviors in Europe and America, e.g. IDUs, men who have sex with men (MSM), and is more commonly transmitted through IDU [36, 43–45]. It is likely that HCV subtype 1a is being efficiently circulated through IDU as a shared risk behavior for both HIV and HCV transmission. In HIV-infected individuals, spontaneous clearance of acute HCV infection is rare; therefore it is likely that most of the HIV-HCV coinfection patients in our study were infected with HCV genotype 1, which was not subsequently cleared [44]. Subtype 1b was predominant in acutely infected patients in our study (28.9%; 93/328). Subtype 1b is also the predominant subtype in Vietnam’s neighboring countries, accounting for 66% of HCV infection in China, 64.4% in Japan, and 66% in South Korea [46–48], and is also a major subtype in Europe [46]. HCV subtype 1b has been shown in the 1990s to be associated with blood transfusion and unsafe medical injections [49]. It might be possible that genotype 1b has introduced to Vietnam from neighboring countries and subsequently spread in the country via transfusion or unsafe medical practices or population movement.

Genotype 6 was the second most common genotype across all study groups. Genotype 6 is the most genetically diverse among HCV genotypes and is known to contain the highest prevalence of preexisting variants conferring resistance to DAAs [35, 50]. It has been hypothesized that genotype 6 has evolved from the Vietnam Mekong Delta region [22, 23, 41]. One third of the viral strains in our study was genotype 6 including six subtypes demonstrating its diversity and abundance in southern Vietnam. The most predominant subtypes were 6a (18.3%; (59/322) followed by 6e (12.1%; 39/322). The prevalence of genotype 6 as well as subtype 6e was significantly higher in chronically-infected HCV mono-infection patients; one might speculate that this is due to poor spontaneous clearance of acute HCV infection with genotype 6. The prevalence of genotype 6 was significantly lower in HIV-HCV coinfection patients (specifically subtype 6e). The reason for such difference is not known; however, possible explanations include less exposure of HIV-infected patients to genotype 6 virus or poor transmission potential of genotype 6 in HIV-infected patients. It has been hypothesized that genotype 6e is indigenous to Vietnam and is subsequently spread to neighboring countries via drug trafficking routes [50]. Subtype 6h, 6k, 6l, and 6p were also identified in a limited number of patients; this supports the earlier hypothesis of the long endemic circulation of genotype 6 in Vietnam [23].

Genotype 2 viral strains were identified in 7.5% (24/322) patients. This includes 13 strains of subtype 2a, 2 strains of 2b, and 9 strains of 2m. It has been hypothesized that subtype 2m originates exclusively in Vietnam as it is not identified elsewhere [51]. HCV genotype 2 had their ancestral origin in Africa and was brought outside Africa by European explorers. Since Vietnam was a French colony in the 18^th^ century; it is possible that the genotype 2 was introduced to the country during the French colonization time [42].

Data on HCV subtype diversity is scanty in Vietnam as most diagnostic kits are not designed to accurately determine the extensive and evolving HCV subtypes. Most clinical laboratories use HCV kits using 5’ UTR region as target for genotyping. It has been reported earlier that the 5’UTR based genotyping has poor discriminatory power to differentiate between Genotype 1 and 6 [52]. Furthermore, the subtypes are evolving due to continuous generation of quasispecies within an infected individual. 15% of the viral strains in our study had discordant genotype when different RNA regions (5’UTR, Core or NS5B) were used for genotyping. However, the majority of the isolates having discordant genotypes belong to genotype 6e. The 5’ UTR region of genotype 1 and 6 have high similarity, and our data further demonstrate that in the majority of genotype 6e isolates, the 5’UTR sequence cannot discriminate between Genotype 1a, 1b and 6e. Regions with high prevalence of genotype 6 viral strains should consider NS5B sequence based genotyping methods.

The presence of recombinant/mixed strains in HCV patients has been reported earlier [53, 54]. Based on the genotype/subtype difference between core and NS5B sequences, 2.2% (7/322) of the viral strains in our study had evidence of mixed/recombinant HCV infection. All mixed/recombinant strains were between genotype 1 and 6; the major genotype in Vietnam. Among these seven patients, 71.5% (5/7) were HIV-HCV co-infected and had history of IDU. The presence of recombinant/mixed strains in a single individual may be due to multiple sequential infections with different genotype/subtype strains or due to super infection with a different genotype/subtype or infection with a mixed genotype viral strains. However, further investigation is necessary to characterize these recombinant strains. The diversity of the viral strains from acutely-infected patients was higher than in chronically-infected patients. It is possible that the high diversity of the viral strains in acute infection are facilitated by the active replication phase that is not influenced by host immunogenetic factors. Next generation sequencing and analysis of quasispecies during acute infection is necessary to understand the extent of diversity as well as the factors that influence viral evolution within host.

We compared HCV RNA and liver enzyme (ALT, AST, albumin, bilirubin, GGT, AFP) levels in acute, HCV mono-infection, and HIV-HCV coinfection. As expected, HCV RNA and liver enzyme levels were higher in acutely-infected patients. HCV RNA and liver enzyme levels in acute, HCV mono-infection, and HIV-HCV coinfection were similar among different genotypes. We have limited number of genotypes in our study and further studies with more genotypes are necessary to explore the hepatic pathogenic potential of different HCV genotypes.

A difference in distribution of HCV genotypes was noted in Central and Southern Vietnam. While genotype 1 was common in both regions, genotype 6 was relatively common in Southern region. This is consistent with a recent report describing a predominance of genotype 6 (52.97%; 125/236) in blood donors and patients with liver diseases in Ho Chi Minh City [23]. The prevalence of genotype 3 was significantly high in central Vietnam especially in the Central Highlands sub-region. In the South East and Mekong Delta sub regions, genotype 2 constitutes 10% and 7% of the viral strains respectively. Further epidemiological studies are essential to understand the underlying reasons for the difference in geographic distribution of HCV genotypes in Vietnam. Knowledge of different risk behaviors of vulnerable populations and of modes of transmission of the circulating viral strains in these sub regions will inform public health prevention strategies.

Prior to the development of DAAs, IL-28B SNP typing was used to predict response to interferon based therapy for HCV patients. Although there is a strong relationship between SNP at or near the IL-28B gene and the SVR with pegINF/RBV treatment for chronic hepatitis C [55], routine testing is not recommended in HCV management guidelines in Vietnam [56]. This is likely due to cost associated with IL28B SNP detection and lack of available technologies. We explored the association of IL28 genotype with acute infection and found no significant difference between in IL28B SNP (rs12979860 and rs8099917) between the non-HCV-infected control group and the acutely-infected patients. However, we identified significantly higher prevalence of CC allele at rs12979860 between acute and chronic HCV infection groups. This suggests that CC allele might be favorable for spontaneous clearance of infection after acute HCV infection [18]. Besides this, a recent study in Thailand has shown that CC allele is associated with higher SVR (84.2% vs. 59.5%, *p*<0.001) in chronically-infected patients treated with PegINF/RBV [57].

Our data have limitations; namely, patients were recruited from provincial hospitals and may not be representative of acute HCV infection in Vietnam. Generally, patients visit their nearest medical facilities including private practitioners and district hospitals. Patients are generally referred to provincial hospitals only if they have severe disease or are living near provincial hospitals. The spatial analysis in our study could be biased by hospital referral practice. Additionally, the referral of patients to provincial hospitals is influenced by several factors including the socioeconomic status of the patient. Data on chronic HCV infection may be biased as patients who were cured after treatment were not available. The sample size for the IL28B SNP analysis might not be robust enough to draw any conclusion. Our data on viral diversity might benefit from further analysis using next generation sequencing and analysis of quasispecies to understand the genetic evolution within host and population.

In summary, we report a large study on HCV genotype and subtype, including for the first time data on acute HCV infection in Vietnam. Our data suggests that the genotype 1a and 1b is common in Vietnam. Genotype 6 is the second most common genotype and is more prevalent in chronic HCV mono-infection patients. Subtype 6e infection is often misdiagnosed as 1a or 1b using commercial genotyping assays; therefore, correct determination of genotype using NS5B sequence is essential for evaluating the success of DAAs in geographic regions where HCV genotype 6 is prevalent.

## Supporting information

S1 Table Geolocation (region and sub region) distribution of 355 patients enrolled in the four studies. (DOCX)

S2 Table Analysis of homology between 5’UTR, core and NS5B region with reference sequence of 7 HCV viral strains. These strains showed discordant genotype when Core and NS5B sequence was used individually for genotype determination.
(DOCX)

S3 Table HCV viral load and liver enzyme profile of acute, chronic HCV mono-infection and HIV-HCV coinfected patients and among different genotypes within each groups. (DOCX)
