# Supplementary material for: Differential prevalence and geographic distribution of hepatitis C virus genotypes in acute and chronic hepatitis C patients in Vietnam
Source: PLoS One. 2019 Mar 13;14(3):e0212734. doi: 10.1371/journal.pone.0212734 (PMC6415813; doi:10.1371/journal.pone.0212734)
Supplement: S1 Table — (DOCX) [file pone.0212734.s001.docx]

S1 Table: Geolocation (region and sub region) distribution of 355 patients enrolled in the four studies.

| Region | Sub region | All patient  100% (355) | Acute infection 54.6% (194) | Chronic infection  22.8% (81) | HIV-HCV Coinfection 22.5% (80) |
| --- | --- | --- | --- | --- | --- |
|  |  | % (n) | % (n) | % (n) | % (n) |
| Southern region | | 88.7 (315) | 80.9 (157) | 100.0 (81) | 96.2 (77) |
|  | South East | 60.5 (215) | 32.8 (63) | 100.0 (81) | 88.8 (71) |
|  | Mekong Delta | 28.1 (100) | 49.0 (94) | 0.0 (0) | 7.5 (6) |
| Central region | | 10.1 (36) | 17.0 (33) | 0.0 (0) | 3.8 (3) |
|  | North Central Coast | 1.4 (5) | 2.6 (5) | 0.0 (0) | 0.0 (0) |
|  | South Central Coast | 4.8 (17) | 7.3 (14) | 0.0 (0) | 3.8 (3) |
|  | Central Highlands | 3.9 (14) | 7.3 (14) | 0.0 (0) | 0.0 (0) |
| Northern region | | 0.6 (2) | 1.0 (2) | 0.0 (0) | 0.0 (0) |
|  | North East | 0.6 (2) | 1.0 (2) | 0.0 (0) | 0.0 (0) |
