# Supplementary material for: Differential prevalence and geographic distribution of hepatitis C virus genotypes in acute and chronic hepatitis C patients in Vietnam
Source: PLoS One. 2019 Mar 13;14(3):e0212734. doi: 10.1371/journal.pone.0212734 (PMC6415813; doi:10.1371/journal.pone.0212734)
Supplement: S2 Table — These strains showed discordant genotype when Core and NS5B sequence was used individually for genotype determination. (DOCX) [file pone.0212734.s002.docx]

S2 Table: Analysis of homology between 5’UTR, core and NS5B region with reference sequence of 7 HCV viral strains. These strains showed discordant genotype when 5’UTR, Core and NS5B sequence was used individually for genotype determination.

|  |  | % similarity with | | |
| --- | --- | --- | --- | --- |
| HCV isolate number (assigned genotype and subtype) | Reference sequence genotype | 5UTR | Core | NS5B |
| 10-C-0083 (6e)**^φ^** | Subtype 1b | 99.6 | 96.3 | **69.9** |
|  | Subtype 6e | 99.8 | 86.2 | 83.4 |
| 01EI-03-408 (1a) **^φ^** | Subtype 1a | 97.6 | 87.4 | 90.9 |
|  | Subtype 6e | 98.0 | 91.0 | **66.3** |
| VHA_8 (1b) **^φ^** | Subtype 1b | 99.8 | 87.0 | 90.6 |
|  | Subtype 6a | 97.9 | 96.1 | **68.4** |
| VHA_17 (1b) **^φ^** | Subtype 1b | 99.0 | 85.8 | 93.8 |
|  | Subtype 6e | 99.0 | 95.6 | **66.2** |
| VHA_64 (1a) **^φ^** | Subtype 1a | 99.4 | 85.6 | 82.6 |
|  | Subtype 6e | 97.5 | 88.0 | **68.3** |
| VHA_62 (1b) **^φ^** | Subtype 1b | 99.8 | 89.0 | 91.7 |
|  | Subtype 6a | 97.9 | 94.2 | **67.4** |
| VHA_18 (6a)* | Subtype 1b | 94.5 | 87.1 | 92.7 |
|  | Subtype 6a | 96.4 | 96.2 | **67.5** |

**^φ^** Discordant between core and 5” UTR and NS5B *Discordant between NS5B and core and 5UTR region.
