# Supplementary material for: Differential prevalence and geographic distribution of hepatitis C virus genotypes in acute and chronic hepatitis C patients in Vietnam
Source: PLoS One. 2019 Mar 13;14(3):e0212734. doi: 10.1371/journal.pone.0212734 (PMC6415813; doi:10.1371/journal.pone.0212734)
Supplement: S3 Table — (DOCX) [file pone.0212734.s003.docx]

S3 Table: HCV viral load and liver enzyme profile of acute, chronic and HIV-HCV coifected patients and among different genotypes within each groups (acute, chronic and HIV-HCV coinfection).

|  | **All patient** |  |  |  |  |  |
| --- | --- | --- | --- | --- | --- | --- |
|  | **All patient** | **Acute infection** | **Chronic infection** | **HIV-HCV coinfection** | **p** | **p** |
| Variable | **N=322** | **N=161** | **N=81** | **N=80** | **(Acute VS** | **(Chronic VS** |
|  | **mean ± SD** | **mean ± SD** | **mean ± SD** | **mean ± SD** | **Chronic)** | **HIV-HCV coinfection)** |
| Creatinine (µmol/L) | 83.1 ± 36.2 | 84.2 ± 51.1 | 82.7 ± 17.0 | 81.9 ± 14.5 | 0.001 | 0.492 |
| AST_SGOT (U/L) | 426.3 ± 635.0 | 760.5 ± 728.6 | 70.4 ± 54.6 | 43.3 ± 16.2 | 0.000*** | 0.000*** |
| ALT_SGPT (U/L) | 520.9 ± 698.2 | 944.4 ± 740.1 | 67.2 ± 69.3 | 38.6 ± 17.6 | 0.000*** | 0.000*** |
| Albumin (g/L) | 42.0 ± 23.6 | 43.5 ± 35.9 | 41.1 ± 4.1 | 40.7 ± 5.8 | 0.185 | 0.009** |
| Bilirubin TT (µmol/L) | 87.1 ± 99.6 | 150.2 ± 95.4 | 14.0 ± 9.8 | 7.6 ± 5.7 | 0.000*** | 0.241 |
| Glucose(mmol/L) | 7.5 ± 16.4 | 10.9 ± 25.7 | 5.8 ± 1.6 | 4.9 ± 1.1 | 0.001 | 0.196 |
| HCV Viral load (IU/ml)_log5 | 245.4 ± 708.6 | 500.1 ± 1085.2 | 48.4 ± 100.8 | 136.2 ± 246.4 | 0.000*** | 0.000*** |
| GGT (U/L) | 233.9 ± 299.8 | 391.8 ± 347.3 | 72.2 ± 84.5 | 0.0 ± 0.0 | 0.000*** | |
| AFP (ng/ml) | 9.6 ± 40.7 | 0.0 ± 0.0 | 7.0 ± 14.3 | 12.3 ± 56.0 |  | 0.128 |
| * p=0.01-0.05; ** p<0.001-0.01; *** p<0.001 | | | | | | |

|  | **All patient** |  |  |  |  |
| --- | --- | --- | --- | --- | --- |
| Variable | Genotype 1 | Genotype 2 | Genotype 3 | Genotype 6 | p |
|  | N=182 | N=24 | N=7 | N=109 |  |
|  | mean ± SD | mean ± SD | mean ± SD | mean ± SD |  |
| Creatinine (µmol/L) | 84.8 ± 32.1 | 76.5 ± 18.2 | 120.2 ± 87.0 | 75.8 ± 15.8 | 0.003** |
| AST_SGOT (U/L) | 393.5 ± 550.5 | 446.6 ± 438.4 | 1302.2 ± 2451.1 | 407.0 ± 495.7 | 0.003** |
| ALT_SGPT (U/L) | 482.3 ± 653.8 | 598.8 ± 601.5 | 1166.9 ± 1942.7 | 517.3 ± 620.0 | 0.075 |
| Albumin (g/L) | 40.7 ± 5.0 | 41.4 ± 5.0 | 38.5 ± 7.4 | 39.5 ± 4.6 | 0.313 |
| Bilirubin TT (µmol/L) | 82.5 ± 107.9 | 96.5 ± 83.7 | 121.8 ± 96.6 | 90.8 ± 88.3 | 0.724 |
| Glucose(mmol/L) | 8.3 ± 21.6 | 8.3 ± 6.1 | 8.1 ± 2.9 | 6.0 ± 2.4 | 0.844 |
| HCV Viral load (IU/ml)_log5 | 229.7 ± 480.8 | 243.5 ± 504.2 | 13.6 ± 17.7 | 292.6 ± 1054.9 | 0.811 |
| GGT (U/L) | 232.5 ± 293.6 | 320.1 ± 416.9 | 177.8 ± 39.4 | 210.2 ± 276.3 | 0.597 |
| AFP (ng/ml) | 12.3 ± 50.2 | 1.8 ± 3.2 | 1.3 ± 0.0 | 5.8 ± 15.8 | 0.744 |
|  |  |  |  |  |  |
|  |  |  |  |  |  |
|  | **Acute infection** |  |  |  |  |
| Variable | Genotype 1 | Genotype 2 | Genotype 3 | Genotype 6 | p |
|  | N=82 | N=16 | N=6 | N=57 |  |
|  | mean ± SD | mean ± SD | mean ± SD | mean ± SD |  |
| Creatinine (µmol/L) | 88.2 ± 47.1 | 72.0 ± 19.6 | 127.0 ± 98.9 | 70.5 ± 17.3 | 0.037* |
| AST_SGOT (U/L) | 794.0 ± 605.0 | 557.0 ± 447.0 | 1513.0 ± 2615.0 | 705.0 ± 507.0 | 0.046* |
| ALT_SGPT (U/L) | 993.0 ± 672.0 | 789.0 ± 600.0 | 1354.0 ± 2058.0 | 906.0 ± 605.0 | 0.266 |
| Albumin (g/L) | 40.0 ± 5.0 | 40.0 ± 5.0 | 36.0 ± 7.0 | 39.0 ± 4.0 | 0.744 |
| Bilirubin TT (µmol/L) | 162.8 ± 110.4 | 114.6 ± 80.0 | 144.6 ± 88.0 | 147.1 ± 73.8 | 0.469 |
| Glucose(mmol/L) | 16.0 ± 39.0 | 9.0 ± 6.8 | 8.8 ± 2.8 | 6.7 ± 3.1 | 0.703 |
| HCV Viral load (IU/ml)_log5 | 478.1 ± 709.5 | 402.9 ± 624.2 | 7.7 ± 13.8 | 661.4 ± 1704.8 | 0.675 |
| GGT (U/L) | 392.9 ± 342.1 | 505.2 ± 478.4 | 177.8 ± 39.4 | 378.0 ± 327.1 | 0.613 |
|  |  |  |  |  |  |
|  | **Chronic mono-infection** | |  |  |  |
| Variable | Genotype 1 | Genotype 2 |  | Genotype 6 | p |
|  | N=38 | N=7 |  | N=36 |  |
|  | mean ± SD | mean ± SD |  | mean ± SD |  |
| Creatinine (µmol/L) | 85.7 ± 21.0 | 85.8 ± 14.1 |  | 79.0 ± 12.2 | 0.326 |
| AST_SGOT (U/L) | 79.0 ± 64.0 | 93.0 ± 74.0 |  | 57.0 ± 36.0 | 0.175 |
| ALT_SGPT (U/L) | 76.0 ± 77.0 | 101.0 ± 141.0 |  | 53.0 ± 37.0 | 0.22 |
| Albumin (g/L) | 42.0 ± 5.0 | 42.0 ± 3.0 |  | 40.0 ± 3.0 | 0.37 |
| Bilirubin TT (µmol/L) | 16.7 ± 12.9 | 7.4 ± 1.1 |  | 11.7 ± 3.6 | 0.168 |
| Glucose(mmol/L) | 5.7 ± 1.5 | 6.9 ± 0.4 |  | 5.7 ± 1.7 | 0.582 |
| HCV Viral load (IU/ml)_log5 | 48.4 ± 80.2 | 23.4 ± 49.9 |  | 53.2 ± 125.9 | 0.779 |
| GGT (U/L) | 76.4 ± 88.6 | 82.1 ± 109.3 |  | 65.7 ± 76.7 | 0.821 |
| AFP (ng/ml) | 9.7 ± 14.9 | 1.9 ± 3.5 |  | 5.1 ± 14.6 | 0.234 |
|  |  |  |  |  |  |
|  | **HIV-HCV coinfection** | |  |  |  |
| Variable | Genotype 1 | Genotype 2 | Genotype 3 | Genotype 6 | p |
|  | N=62 | N=1 | N=1 | N=16 |  |
|  | mean ± SD | mean ± SD | mean ± SD | mean ± SD |  |
| Creatinine (µmol/L) | 81.4 ± 15.0 | 80.0 ± 0.0 | 92.8 ± 0.0 | 83.2 ± 13.7 | 0.856 |
| AST_SGOT (U/L) | 44.0 ± 16.0 | 0.0 ± 0.0 | 40.0 ± 0.0 | 42.0 ± 16.0 | 0.906 |
| ALT_SGPT (U/L) | 40.0 ± 18.0 | 48.0 ± 0.0 | 46.0 ± 0.0 | 34.0 ± 18.0 | 0.586 |
| Albumin (g/L) | 41.0 ± 5.0 | 51.0 ± 0.0 | 45.0 ± 0.0 | 39.0 ± 7.0 | 0.223 |
| Bilirubin TT (µmol/L) | 7.7 ± 6.3 | 4.3 ± 0.0 | 7.6 ± 0.0 | 7.5 ± 3.3 | 0.951 |
| Glucose(mmol/L) | 4.9 ± 1.1 | 4.8 ± 0.0 | 5.4 ± 0.0 | 5.2 ± 0.9 | 0.685 |
| HCV Viral load (IU/ml)_log5 | 132.6 ± 254.4 | 31.4 ± 0.0 | 37.0 ± 0.0 | 162.9 ± 233.3 | 0.913 |
| AFP (ng/ml) | 14.0 ± 62.9 | 1.2 ± 0.0 | 1.3 ± 0.0 | 7.4 ± 18.8 | 0.969 |

* p=0.01-0.05; ** p<0.001-0.01; *** p<0.001
